# Changes in Upper-Body Muscular Strength and Power in Paralympic Swimmers: Effects of Training Confinement during the COVID-19 Pandemic

**DOI:** 10.3390/ijerph19095382

**Published:** 2022-04-28

**Authors:** Luca Cavaggioni, Alessio Rossi, Massimiliano Tosin, Raffaele Scurati, Giovanni Michielon, Giampietro Alberti, Giampiero Merati, Damiano Formenti, Athos Trecroci

**Affiliations:** 1 Obesity Unit—Laboratory of Nutrition and Obesity Research, Department of Endocrine and Metabolic Diseases, IRCCS Istituto Auxologico Italiano, 20145 Milan, Italy; 2 Department of Biomedical Sciences for Health, Università degli Studi di Milano, 20133 Milan, Italy; raffaele.scurati@unimi.it (R.S.); giovanni.michielon@unimi.it (G.M.); giampietro.alberti@unimi.it (G.A.); athos.trecroci@unimi.it (A.T.); 3 Department of Computer Science, University of Pisa, 56127 Pisa, Italy; alessio.rossi2@gmail.com; 4 Italian Paralympic Swimming Federation, 00144 Rome, Italy; lombardia@finp.it; 5 Department of Biotechnology and Life Sciences, University of Insubria, 21100 Varese, Italy; gpmerati@dongnocchi.it (G.M.); damiano.formenti@uninsubria.it (D.F.); 6 IRCCS Fondazione don Carlo Gnocchi, 20148 Milan, Italy

**Keywords:** Paralympic swimming, dry-land training, load-velocity profile, power, strength, disability, exercise

## Abstract

The aim of this case series was to evaluate the effectiveness of a dry-land home-training program conducted during the COVID-19 pandemic period in Paralympic swimmers. Previous evidence showed the importance of muscular strength and power training for Paralympic swimmers due to the positive relationship between severity of impairment, swimming technique and biomechanics parameters. Specifically, we aimed to analyze: (i) the effects of a customized training regime conducted pre, during and post restrictions on upper-body muscular strength and power (one repetition maximum, mean propulsive velocity, and mean relative propulsive power) compared to a regular gym-based program; (ii) the associations between mean propulsive velocity and load during two upper body exercises in order to estimate the one repetition maximum. Four elite Paralympic swimmers were retrospectively analyzed in upper-body muscular strength, mean propulsive velocity and mean relative propulsive power in bench press and lat pull-down exercises at three time points: T0 (prior the Lockdown period), T1 (immediately after the Lockdown confinement), T2 (sixteen weeks after returning to gym training). Our findings suggest a *very likely* decrement in one repetition maximum, mean propulsive velocity, and mean relative propulsive power during the Lockdown period compared with the T0 period with a subsequent *very likely* increment in one repetition maximum after returning to gym training (T2) compared with the lockdown period (T0). Conversely, mean relative propulsive power showed an *unclear* improvement in all athletes in T2 compared with T1. These results were also corroborated by the Friedman’s test followed by the Dunn’s pairwise comparison that mainly showed a decrement from T0 to T1 (*p* < 0.05). At the same time, it appears that muscle strength and power could be rapidly restored close to the pre-lockdown levels following an adequate training program in the gym, albeit without significance (*p* > 0.05). Finally, the close relationship between mean propulsive velocity and load in bench press and lat pull-down exercises was also confirmed in para swimming, making a possible estimation of one repetition maximum.

## 1. Introduction

The recent widespread of the global Pandemic COVID-19 modified habits and behaviors of common people and athletes at different levels due to “SARS-CoV-2” virus diffusion. In this scenario, to limit contagion, many preventive measures (e.g., social distancing, hygiene protocols, and isolation) have been regularly adopted by governments and local authorities, as well as COVID-19 vaccines to guarantee protection from a severe disease [1]. In Italy, the decree of the Italian Prime Minister on March 2020 imposed general stay-at-home restrictions over the entire country allowing limited, documented, essentials displacements (e.g., for health or basic commodities) [2]. In addition, many business and sport activities (i.e., gyms or fitness facilities) have been temporarily closed for several months [3]. In fact, athletes of different disciplines (both team and individual sports) had forced to stop from competitive events (e.g., from the National championship to the Olympic-Paralympic Games in Tokyo) that were postponed or cancelled [4]. Nevertheless, national-level athletes had the possibility to continue in their regular training program even during the lockdown period.

Similarly to what occurs during an injury, a sudden interruption from regular training longer than four weeks (e.g., due to forced confinement in the COVID-19 pandemic) can lead athletes to a progressive detraining in various cardio-pulmonary (e.g., VO_2_max, maximal stroke volume, cardiac output and artero-venous O_2_ difference) and musculoskeletal (e.g., muscle mass, muscle strength and power) parameters [5,6]. Indeed, to counteract the negative effects of home confinement, previous papers recommended an active lifestyle focused on a regular home-based physical activity practice [7,8] both for common people and athletes. Recent evidence provided direct support for this recommendation suggesting that a home-based physical routine executed in the COVID-19 confinement should be structured to maintain levels of muscular strength, postural control, core-stability, and body balance parameters [9].

Nevertheless, the effects of COVID-19 pandemic restrictions on muscular strength variables have not been investigated in Paralympic athletes yet. As a matter of fact, they often struggle to train alone compared to able-bodied athletes. In this context, a study conducted on competitive swimmers showed that strength and power-related variables were markedly reduced after 4 weeks of inactivity [10]. Moreover, previous evidence showed the importance of muscular strength and power training for Paralympic swimmers due to the positive relationship between severity of impairment and swimming technique [11,12,13,14].

Therefore, the aim of this case series was to evaluate the effects of training restrictions on upper-body muscular strength and power in Paralympic swimmers comparing their level status pre, during, and post COVID-19 confinement period. Moreover, a second aim of this study was to assess a possible association between the mean propulsive velocity and load during upper body exercises. Due to the decrement in training parameters related to the home-based training and the impossibility to use gym facilities, we hypothesized a decrement in muscular strength and power performances during COVID-19 confinement. However, these decrements could be reversed in the subsequent retraining period in the gym.

## 2. Materials and Methods

### 2.1. Participants

A case series on Paralympic athletes (3 males and 1 female; age = 24.3 ± 3.8 years; height = 1.73 ± 0.20 m, weight = 72.2 ± 13.1 kg, mean ± SD) was conducted. A total of 4 elite Paralympic swimmers were recruited from Northern Italy (they belong to the same country) and voluntarily participated in this study. The four athletes participated at major international (i.e., European Championship, World Championship and Paralympic Games) or national competitions (i.e., Italian Championships) at least once. They had a minimum of three years background in competitive swimming and reached the podium in one of the above-mentioned competitions. As for the para swimming functional classes, athlete 1 (A1) has a cerebral palsy and belonged to the S5 class, athlete 2 (A2) has a hereditary spastic paraparesis and belonged to the S6 class, athlete 3 (A3) has a lower limb deficiency and belonged to the S9 class and, finally, athlete 4 (A4) has lower limb amputation and belonged to the S8 class.

The four athletes provided a written informed consent before starting the investigation. All procedures were in accordance with the Declaration of Helsinki and the ethics committee of the local University approved the investigation.

### 2.2. Procedures

Athletes were tested at three time points: two weeks prior to COVID-19 lockdown (T0), following the home-based training due to the restrictions’ period (from March 2020 to September 2020) (T1) and sixteen weeks after returning to a regular gym-training (T2). Each testing session was conducted in the same indoor gym with similar conditions (temperature 21–24 °C, relative humidity 42–51%) and at the same time of day (3.00 p.m. to 5.00 p.m.). All participants were requested to abstain from consuming alcoholic or caffeinated drinks before the testing day. For the intervention period, each athlete lived in their own home and regarding the nutritional aspects they followed a balanced mixed diet consisting of a variety of nutrients in carbohydrate intake and high-biological-value protein, which was sufficient to meet the micro and macronutrients required.

### 2.3. Muscular Strength and Power Assessment

Lat pull-down and bench press exercises were performed following the protocol proposed for Paralympic judokas [15] to determine the one repetition maximum, mean propulsive velocity of the barbell, the mean propulsive velocity obtained close to the one repetition maximum, and the mean relative propulsive power. To account for the differences in body mass between athletes, power values were normalized dividing their absolute power by the body mass (W·kg^−1^). These tests and parameters were selected because previous studies found that the a positive association between dry-land exercises with the force expressed during swimming performance [16,17]. In detail, each athlete warmed up for 12 min by arm crank exercise at a self-selected pace (low intensity), dynamic stretching and 10–15 repetitions of bench press or lat pull-down with 50% 1RM. Participants were asked to perform 3 repetitions at maximal speed for each load, starting at a criterion load of 30% of their body mass. The load progressively increased by 5% in each set until a decrease of 0.6 m/s occurred in mean propulsive velocity. Swimmers had to lower the bar in a controlled manner until it touched the chest. Then, after the command “start”, they moved the bar as fast as possible. Each set was interspersed by 5 min of rest to allow a complete recovery. The highest load that each athlete could lift for at least one time was considered as the actual one repetition maximum. To calculate mean relative propulsive power _l_ and mean propulsive velocity, a wearable wireless sensor (Beast sensor, Beast Technologies Srl, Milan, Italy), for which validity and reliability (ICC = 0.98) was previously established [18,19], was attached at the barbell using a built-in magnet. The Beast sensor is composed of a 3-axial accelerometer, magnetometer, and gyroscope (15.2 cm^3^ of volume, 38 g of weight) that measures speed with a sample rate of 50 Hz and connected to a portable tablet through Bluetooth 4.0 (Android system). The mean propulsive velocity and mean relative propulsive power output were transferred in real time to a dedicated software (Beast sensor v. 2.3.7, Beast Technologies Srl). The entire testing session was performed in an indoor gym.

### 2.4. Experimental Protocol

Prior to the mandatory COVID-19 restriction period, swimmers were attending a regular weekly pool training consisting of five sessions and three resistance dry-land training sessions with the aim to increase muscular strength (intensity 70–80% 1RM), flexibility, and postural control performing whole-body movements using free weights or cable machine equipment [20,21]. Regarding the training routine, all of the regular weekly training consisted of eight sessions (including aerobic, anaerobic, and technical components) and three resistance-training sessions, throughout the experimental period (from October to August), divided into two major macrocycles (October to March and March to August), with regular monitoring of the daily training load. The first macrocycle was mainly characterized by developing a proper breathing pattern awareness, from static to dynamic stability and from general to maximum strength development. Subsequently, the second macrocycle was even more sport specific, focused on specific breathing pattern contribution during swimming, specific static and dynamic core stabilization stimuli, and specific muscular power and power endurance development. From Monday to Friday, they performed one swimming session per day, three dry-land training sessions (Monday, Wednesday, Friday), and one swimming session on Saturday. A typical pool training session consisted of swimming technique or coordination exercises, and aerobic (aerobic capacity and aerobic power) or anaerobic (speed, lactic capacity, and power) drills. All training procedures were prescribed and monitored by their swimming coaching staff. During the confinement period, no access to gym facilities was permitted. However, to attenuate the expected decay in physical fitness caused by the restriction, the athletes followed a three-weekly frequency home-based resistance training program. The athletes also performed bodyweight exercises with elastic-band or common household items as external resistance in standing or sitting positions (pushing exercises as push-up or push-press, pulling exercises as rows with bands, squatting or hip-hinging exercises and core-muscles isometric exercises). The swimming coaching staff monitored their training through virtual video communication. During the lockdown period, a decree of the Italian Prime Minister allowed the national-level recognized athletes to participate in training sessions; thus, the athletes began to perform their regular swimming sessions five times a week (90 min each), during which regular safety procedures were observed (e.g., social distancing, temperature assessment, PCR testing, protective devices, vaccination). After the restriction period (28 weeks), all athletes returned to their regular gym training, and at this time point they were firstly assessed (T1) and then continued to observe their pre-lockdown dry-land training program respecting a three-week frequency. To avoid any overuse injuries, the intensity of training was progressively increased from 50–60% 1RM in the first four weeks, to 60–70% 1RM in the middle six weeks until reaching 70–80% 1RM in the last six weeks, just before the final assessment (T2).

### 2.5. Data Analysis

To detect individual changes over time (from T0 to T1 and from T1 to T2), magnitude-based inference (MBI) was used. MBI allows users to qualify and quantify the interpretation of the mean difference linked to an individual smallest worthwhile change (SWC [1/3 of the performance coefficient of variation]). The chance that the change between measurements occurs was assessed qualitatively as follows: *very likely*, *clear* and *unclear*. To investigate potential group-differences between the three time points for each variable, Friedman’s test was conducted. In case of significance, Dunn’s test was used for pairwise comparisons. Finally, the relationship between bar velocity and load in both exercises were determined using Pearson’s correlation coefficient, whereas the error of the relationship between the one repetition maximum values and mean propulsive velocity (linear regression analysis) was assessed by root-mean-squared error (RMSE). The interpretation of the magnitude of the correlation was performed using the following scale: <0.1, trivial; 0.1–0.29, small; 0.3–0.49, moderate; 0.5–0.69, large; 0.7–0.9, very large; >0.9, nearly perfect [22]. The statistical analysis was performed using Python 3.8 language programming, setting the statistical significance at *p* < 0.05, whereas the individual variations over time was computed by using the magnitude-based inference approach (MBI) for individual trend on a customized spreadsheet available at www.sportsci.org/index.html (accessed on 1 February 2021).

## 3. Results

The COVID-19 pandemic restrictions’ regarding limited country displacements allowed us to recruit a total of five eligible athletes, but one athlete dropped out due to an injury that occurred during the intervention period. In light of this, the final sample comprised four individuals who were considered for statistical analysis. No other adverse situations were reported during the study period.

Table 1 highlights the individual changes obtained with the qualitative inference in one repetition maximum, mean propulsive velocity close to the one repetition maximum and mean relative propulsive power outcomes over time. Regarding one repetition maximum, it is possible to appreciate a *very likely* effect in each time point (from T0 to T1 and from T1 to T2) in both lat pull-down and bench press exercises. Moreover, observing the qualitative inference of mean propulsive velocity close to the one repetition maximum parameter, an *unclear* effect was found in all athletes for both exercises over time (from T0 to T1 and from T1 to T2). Finally, mean relative propulsive power showed a *very likely* difference from T0 to T1, whereas an *unclear* effect from T1 to T2 in all swimmers. Specifically, in lat pull-down there was a *substantial* decrease from T0 to T1 in all the athletes and an *unclear* increase from T1 to T2 in A1, A2, and A3. Inversely, A4 showed a *substantial* increase between T1 and T2. Regarding the bench press test, it is possible to highlight a *substantial* decrease from T0 to T1 in all four athletes and an *unclear* increase from T1 to T2 in A1, A2, and A3, whereas A4 demonstrated a *substantial* increase.

Friedman’s test showed significant differences between the three time points for all variables (*p* < 0.05). Specifically, significant decrements of one repetition maximum, mean propulsive velocity and mean relative propulsive power were found from T0 to T1 for both bench press and lat pull-down (*p* < 0.05), except for the mean propulsive velocity in the bench press.

**Figure 1:**
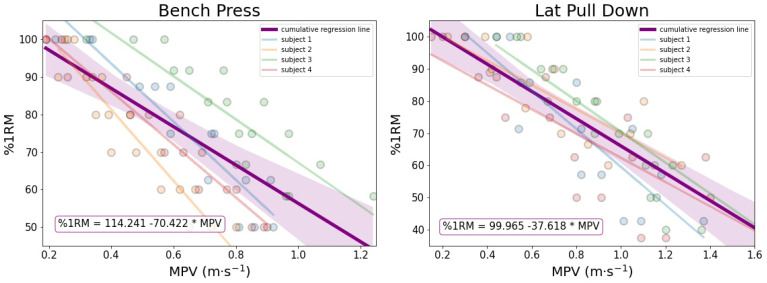
Relationship of all athletes between the percentage of one repetition maximum (%1RM) and the mean propulsive velocity (MPV) in bench press and lat pull-down exercises. Purple lines are the cumulative linear regression lines with 95% confidence intervals of all subjects.

Figure 1 shows the significant relationship existing between the percentage of one repetition maximum and mean propulsive velocity for both exercises. In actual fact, the one repetition maximum percentage in both bench press and lat pull-down shows a strong negative correlation with mean propulsive velocity (r = −0.74, R^2^ = 0.55, *p* < 0.0001, and r = −0.83, R^2^ = 0.69, *p* < 0.0001, respectively). The cumulative equation (for all subjects) that permits to estimate one repetition maximum percentage from mean propulsive velocity is reported in Figure 1.

## 4. Discussion

The aim of this study was to investigate the effects of training restrictions on upper-body muscular strength and power in elite Paralympic swimmers. The main findings of this case series were that maximum strength had a *substantial decrease* from pre-lockdown until the end of the confinement period (i.e., T0 vs. T1) and *substantial increase* in the following period (i.e., T1 vs. T2). Additionally, the mean relative propulsive power showed a *substantial decrease* from T0 to T1, whereas an *unclear* change was detected from T1 to T2. The decrease in neuromuscular parameters in relation to the training confinement due to COVID-19 pandemic was also partially corroborated by Friedman’s test, showing a significant decrease in almost all variables from T0 to T1 (except for the mean propulsive velocity of the bench press). Lastly, it was observed an association between mean propulsive velocity and load that could facilitate the one repetition maximum assessment starting from the simple measurement of the mean propulsive velocity. In actual fact, the slower the velocity, the higher the %1RM is.

The maximum strength and mean relative propulsive power had a slightly reduction during the Lockdown period both in bench press and lat pull-down exercises. Previous evidence showed controversial responses in the neuromuscular performance after lockdown period in able-bodied athletes [17,18]. In actual fact, on one hand, a recent evidence showed a maintenance in one repetition maximum and in countermovement jump height in able-bodied female football players during the lockdown period [23], on the other hand it has been demonstrated a significant reduction in rate of force development, peak power, velocity, and landing peak force in able-bodied futsal players [24]. Unfortunately, no data in Paralympic athletes have been reported in the literature yet. Recent evidence showed a positive association between strength-related outcomes (i.e., tethered force), swimming technique and biomechanics (i.e., stroke length, stroke frequency, swimming speed) and nature of physical impairment (i.e., upper-body amputation) [25,26,27].

Regarding our case series, it is also worth noticing that the regular strength and conditioning program was managed to allow a safe home-training, respecting the individual physical capacity, fitness level, and nature of impairment. From a speculative view, the type of equipment used at home (e.g., bodyweight exercises, elastic band) compared to a gym setting, makes difficult to perform moderate-to-high intensity resistance training, leading to an insufficient training stimulus for a further development in muscular strength and power.

The second remarkable finding of this study was that in the post-restrictions’ period (T2 = sixteen weeks after returning to a regular gym-training) there was a *substantial* increase in one repetition maximum in all athletes compared to the pre-COVID-19 confinement, whereas the mean relative propulsive power parameter showed an *unclear* change. Previous evidence highlighted that at least four-weeks of retraining are needed to recover strength-related variables after a short period of detraining [28]. The upper body strength and power enhanced by neuromuscular adaptations observed in the retraining period thanks to the nature of the training program itself. In fact, it focused mainly on promoting muscle strength (motor unit recruitment, firing rate), flexibility, and postural control using free weights or cable machines allowing a better intensity management.

This dry-land training modality was demonstrated to be sufficient to obtain changes in muscular power, functional performance, and postural control in elite Paralympic swimmers [20,21]. Presumably, the home-based training program performed during the COVID-19 confinement period was able to maintain the level of physical fitness as recently demonstrated in elite able-bodied swimmers [29]. In addition, it was shown that upper-body muscular strength and power are key elements in swimming performance [30] also in Paralympic athletes [11]. In this regard, a more severe impairments could produce a more pronounced asymmetry and a lowered force production compared to a swimmer with a less severe disability [12].

A side finding of the present study was the close association between the mean propulsive velocity and load that was already observed in many other sports [31,32]. Concerning the wide range of sports in the Paralympic arena, the usefulness of mean propulsive velocity were highlighted in wheelchair basketball athletes and visually impaired sprinters [33,34]. However, to the best of our knowledge, this is the first study on Paralympic swimmers. The close relationship between load and bar velocity can help to estimate which percentage of 1RM should be used when the first repetition of a set is performed [31], thus facilitating the one repetition maximum prediction.

In summary, the portrait emerging from our findings was that, on one hand the home-based exercise program was not effective to sustain pre-lockdown one repetition maximum and mean relative propulsive power performances, on the other each swimmer was able to regain the maximum strength, the mean propulsive velocity and the mean relative propulsive power in sixteen weeks after returning to a regular gym training. Lastly, as seen in other Paralympic sports, it was confirmed an association between bar velocity and load in para swimming as well. From a practical viewpoint, athletic trainers and coaches should consider customizing home-based training when an interruption from the regular training routine is expected (i.e., pandemic condition), because it might be helpful for the following retraining phase. Moreover, it should be encouraged to monitor the bar velocity for a better one repetition maximum prediction, as this measure helps to draw a more detailed picture regarding the athlete’s neuromuscular features. This information will assist strength and conditioning coaches with program training decisions (i.e., to implement a specific type of strength quality throughout the swimming season) derived from individual’s strength and power measurements.

The present study has two main limitations that should be considered. The small number of para swimming functional classes involved (S5, S6, S8, S9) and the lack of a control group do not make possible further generalization, also limiting the data interpretations. However, it should be noted that, albeit the low sample size, our findings based on individual changes were also corroborated by a group analysis based on a non-parametric test. Nevertheless, the relatively small statistical power due to our subjects’ characteristics (elite, highly trained athletes) suggest further studies to investigate the same outcomes with larger sample sizes. Finally, the exercise equipment used during the home-based training compared with the gym setting modified the intensity and volume of resistance training programs and might have influenced the final results.

## 5. Conclusions

This case series showed that maximum strength, mean propulsive velocity, and mean relative propulsive power were negatively impacted during the COVID-19 restriction period, despite a home-based training. This may be due to the inadequacy of equipment used at home to maintain strength levels. At the same time, it appears that muscle strength and power could be rapidly restored close to the pre-lockdown levels following an adequate training program in the gym. Moreover, it has been observed a relationship between the bar velocity and load that could facilitate coaches and practitioners in the one repetition maximum prediction. This may be useful for individualizing more detailed dry-land training programs derived from individual’s strength and power measurements. Finally, when an interruption from the regular training routine is needed, it could be helpful to customize home-based programs because of their beneficial effect for the subsequent retraining phase, especially for Paralympic swimmers in which strength and power variables are closely related to technical parameters. However, a reduction in strength and power should be expected during a home-based training period.

## Figures and Tables

**Table 1:** Individual changes overtime regarding strength and power variables in lat pull-down and bench press exercises.

Athlete	Outcome	T0	T1	T2	Percent Chances of	Qualitative Inference
					Substantial Increase/Trivial/Substantial Decrease	
					(T0 vs. T1); (T1 vs. T2)	(T0 vs. T1); (T1 vs. T2)
A1	**Lat pull-down**					
	1RM_Load_ (kg)	88.7	78.1	88.4	0/0/100; 100/0/0	Very likely; Very likely
	1RM_MPV_ (m·s^−1^)	0.5	0.3	0.44	35/18/47; 45/18/37	Unclear; Unclear
	MPP_rel_ (W·kg^−1^)	6.68	4.10	5.03	3/3/94; 68/13/18	Very likely; Unclear
	**Bench Press**					
	1RM_Load_ (kg)	47.7	44.4	47.2	1/1/98; 97/1/2	Very likely; Very likely
	1RM_MPV_ (m·s^−1^)	0.34	0.22	0.33	43/13/44; 44/13/43	Unclear; Unclear
	MPP_rel_ (W·kg^−1^)	4.69	2.56	2.82	4/3/93; 52/13/35	Very likely; Unclear
A2	**Lat pull-down**					
	1RM (kg)	60.7	49.3	52.7	0/0/100; 100/0/0	Very likely; Very likely
	1RM_MPV_ (m·s^−1^)	0.58	0.39	0.22	33/17/50; 34/17/49	Unclear; Unclear
	MPP_rel_ (W·kg^−1^)	5.35	3.62	4.00	3/3/94; 58/16/26	Very likely; Unclear
	**Bench Press**					
	1RM (kg)	56.0	51.8	54.9	0/0/100; 100/0/0	Very likely; Very likely
	1RM_MPV_ (m·s^−1^)	0.28	0.25	0.26	38/18/44; 42/18/40	Unclear; Unclear
	MPP_rel_ (W·kg^−1^)	3.01	2.27	2.61	5/4/91; 71/12/17	Very likely; Unclear
A3	**Lat pulldown**					
	1RM (kg)	129.8	111.3	128.4	0/0/100; 100/0/0	Very likely; Very likely
	1RM_MPV_ (m·s^−1^)	0.55	0.53	0.44	34/26/39; 26/25/49	Unclear; Unclear
	MPP_rel_ (W·kg^−1^)	8.61	8.08	8.10	3/5/91; 40/26/33	Very likely; Unclear
	**Bench Press**					
	1RM (kg)	78.1	70.0	77.4	0/0/100; 100/0/0	Very likely; Very likely
	1RM_MPV_ (m·s^−1^)	0.57	0.32	0.47	32/10/58; 53/11/37	Unclear; Unclear
	MPP_rel_ (W·kg^−1^)	5.85	4.47	4.99	4/2/94; 71/8/21	Very likely; Unclear
A4	**Lat pull-down**					
	1RM (kg)	84.9	79.3	82.9	0/0/100; 100/0/0	Very likely; Very likely
	1RM_MPV_ (m·s^−1^)	0.30	0.15	0.20	3/3/94; 67/16/17	Very likely; Unclear
	MPP_rel_ (W·kg^−1^)	9.17	6.45	7.49	0/0/100; 90/1/9	Very likely; Very likely
	**Bench Press**					
	1RM (kg)	54.0	50.9	53.0	0/0/100; 100/0/0	Very likely; Very likely
	1RM_MPV_ (m·s^−1^)	0.19	0.19	0.24	39/21/39; 66/16/18	Unclear; Unclear
	MPP_rel_ (W·kg^−1^)	3.59	2.95	3.37	0/0/100; 100/0/0	Very likely; Very likely

1RM = one repetition maximum, 1RM_MPV_ = Mean propulsive velocity close to the one repetition maximum, MPP_rel_ = Mean relative propulsive power.

## Data Availability

The data presented in this study are available on request from the corresponding author.
